# Impaired neonatal macrophage phagocytosis is not explained by overproduction of prostaglandin E2

**DOI:** 10.1186/1465-9921-12-155

**Published:** 2011-12-05

**Authors:** Megan N Ballinger, Marc Peters-Golden, Bethany B Moore

**Affiliations:** 1 Department of Internal Medicine, Division of Pulmonary and Critical Care Medicine, The University of Michigan, Ann Arbor, MI USA

**Keywords:** macrophage, host defense, cellular mediators, phagocytosis, lung

## Abstract

**Background:**

Neonates and young infants manifest increased susceptibility to bacterial, viral and fungal lung infections. Previous work has identified a role for eicosanoids in mediating host defense functions of macrophages. This study examines the relationship between alveolar macrophage (AM) host defense and production of lipid mediators during the neonatal period compared to adult AMs.

**Methods:**

AMs were harvested from young (day 7 and day 14) and adult (~10 week) rats. The functionality of these cells was assessed by examining their ability to phagocytose opsonized targets, produce cytokines, eicosanoids and intracellular cAMP measured by enzyme immunoassays, and gene expression of proteins, enzymes and receptors essential for eicosanoid generation and phagocytosis measured by real time RT-PCR.

**Results:**

AMs from young animals (day 7 and 14) were defective in their ability to phagocytose opsonized targets and produce tumor necrosis factor (TNF)- α. In addition, young AMs produce more prostaglandin (PG) E_2_, a suppressor of host defense, and less leukotriene (LT) B_4_, a promoter of host defense. Young AMs express higher levels of enzymes responsible for the production of PGE_2 _and LTB_4_; however, there was no change in the expression of E prostanoid (EP) receptors or LT receptors. Despite the similar EP profiles, young AMs are more responsive to PGE_2 _as evidenced by their increased production of the important second messenger, cyclic AMP. In addition, young AMs express higher levels of PDE3B and lower levels of PDE4C compared to adult AMs. However, even though the young AMs produced a skewed eicosanoid profile, neither the inhibition of PGE_2 _by aspirin nor the addition of exogenous LTB_4 _rescued the defective opsonized phagocytosis. Examination of a receptor responsible for mediating opsonized phagocytosis showed a significant decrease in the gene expression levels of the Fcgamma receptor in young (day 7) AMs compared to adult AMs.

**Conclusion:**

These results suggest that elevated production of PGE_2 _and decreased production of LTB_4 _do not contribute to impaired opsonized macrophage phagocytosis and highlight an important difference between young and adult AMs.

## Background

Lung infections account for more global burden of disease than any other category, including AIDS, cancer, ischemic cardiovascular disease and diarrheal diseases [1]. Due to the risk of infection from a myriad of microorganisms, the lung has developed an efficient host defense system to protect itself. In a naïve, uncompromised host, resident alveolar macrophages (AMs) are thought to be the primary cell involved in the recognition, ingestion, and eventual destruction of pathogens. AMs phagocytose antibody opsonized pathogens via engagement of their Fcγ receptors.

Children, particularly neonates and young infants, have an altered immune system, increasing their susceptibility to infections, many of which involve the lung. Although the total number of AMs in newborn lungs is slightly less than in adults, it is more likely that the functional immaturity of the AMs in neonates results in the enhanced susceptibility to infection [2]. Animal models have shown that young AMs are defective in both opsonized [3] and unopsonized [4-6] phagocytosis and killing [3] compared to AMs from an adult. While it is generally accepted that newborns are more vulnerable to infectious insult it is not known whether this relates to differences in phagocytic receptors on innate immune cells or alterations in soluble mediators.

Secreted mediators are important regulators of the host immune response to infection. Eicosanoids are lipid mediators which are synthesized and secreted by virtually all cell types and are capable of eliciting a wide spectrum of biological responses [7]. Two classes of eicosanoids that mediate host defense function are leukotrienes (LTs) and prostaglandins (PGs). LTs, consisting of both leukotriene B_4 _(LTB_4_) and cysteinyl leukotrienes (LTC_4_, LTD_4_, and LTE_4_), augment bacterial phagocytosis and killing by AMs [8]. LTs exert their antimicrobial functions by ligating different G-protein coupled receptors (GPCRs) known as B leukotriene type (BLT) 1 and 2 receptor and cysteinyl LT type 1 (cysLT1) and 2 receptor. By contrast, PGE_2 _is an important negative regulator of host innate immunity, with the ability to inhibit AM phagocytosis and killing [9]. PGE_2 _signals through four different GPCRs, termed E prostanoid (EP) receptors 1-4, to effect intracellular signaling pathways, such as cyclic AMP (cAMP) [10]. Interestingly, overproduction of PGE_2 _and/or underproduction of LTs have been shown to negatively regulate host defense function in a variety of immunosuppressed states, including: cancer, HIV infection, malnutrition, and stem cell and solid organ transplant recipients [11].

Previous work has examined the role of eicosanoids in regulating host defense function during aging. Thus far, two studies have found elevated PGE_2 _production and decreased LTs by newborn macrophages compared to adults [12,13]. Interestingly, elevated cAMP levels were also found in mononuclear cells isolated from neonates [14]. These studies suggested that the eicosanoid imbalance in neonates could explain the impaired AM function. Our current studies set out to definitively test this hypothesis. Interestingly, while our results verified the eicosanoid imbalance in AMs from young versus adult animals, we could not demonstrate that eicosanoids regulated the function of AMs from young mice. Instead, our results suggest that diminished expression of FcγR may contribute.

## Methods

### Animals

Adult pathogen-free 125 to 150 g female (approximately 10 weeks of age) Wistar rats were obtained from Charles River Laboratories. In addition, lactating dams with either day 7 or day 14 female pups were also obtained from Charles River. Animals were treated according to National Institutes of Health guidelines for the use of experimental animals, with approval of the University of Michigan Committee for the Use and Care of Animals.

### Alveolar macrophage isolation

Resident AMs were harvested as previously described [8] although there were differences in the volumes used to lavage each of the different age groups. A total of 5 ml was used to lavage the day 7, 10 ml for the day 14, and 50 ml for the adult rats.

### Opsonized phagocytosis assay

The ability of AMs to phagocytose an opsonized target was performed as previously described [9]. Briefly, AMs were harvested from each age group and plated at 2 × 10^5 ^cells per well. The following days, AMs were washed and treated with either Aspirin (Sigma), LTB_4 _(Cayman Chemical) or vehicle control for 20 min before the addition of opsonized sheep red blood cells (RBCs) (ICN Pharmaceuticals) at an MOI of 50:1 for 90 min. All non-ingested RBCs were washed away with cold PBS and the amount of internalization was assessed by microcolorometric determination of intracellular hemoglobin.

### Measurement of cytokines, eicosanoids, and intracellular cAMP

AMs from different age groups were harvested and cultured at 1 × 10^6 ^cells per well for 24 h with and without LPS (500 ng, Sigma) or for 1 h with Ca^2+ ^ionophore A23187 (5 μM, Sigma) or for 15 min with PGE_2 _(1 μM, Cayman Chemical). To measure cytokines or eicosanoids, the cell-free supernatants were collected and analyzed by specific ELISA for either TNF-α (R&D systems) or LTB_4 _and PGE_2 _(Cayman Chemical). To measure cAMP, cells were lysed in 0.1 M HCl for 20 min then intracellular cAMP levels were determined by ELISA according to the manufacturer (Assay Designs).

### Semi-quantitative real-time PCR

Semiquantitative real-time PCR was performed on an ABI Prism 7000 thermocycler (Applied Biosystems). Gene-specific primers were designed using Primer Express software (PerkinElmer/PE Applied Biosystems) and are shown in Table 1. Briefly, the reaction mixture contained 300 ng of cDNA, 12.5 μl of SYBR Green PCR Master Mix (Applied Biosystems), and forward and reverse primers at 300 nM. For each experiment, samples were run in triplicate. The average cycle threshold (C_T_) was determined for each rat from a given experiment. Relative gene expression (using the formula 2^-ΔΔCT^) was calculated using the comparative C_T _method, which assesses the difference in gene expression between the gene of interest and an internal standard gene (β-actin).

**Table 1:** List of primers used for measuring gene expression by real time PCR

Gene name	Forward Primer	Reverse Primer
β-actin	CCTAAGGCCAACCGTGAAAA	AGGGACAACACAGCCTGGAT

COX-1	GACCTGCCCCTATGTCTCCTT	TCACGAAGACAGACCCGTCAT

COX-2	GGATCCCCAAGGCACAAATA	CCTCGCTTCTGATCTGTCTTGA

5-LO	GCTGCACAGAGTTGCCTAAGAA	TCTAAACTGAGCTGCCGCTCTA

FLAP	CCTTCGCTGGGCTGATGT	TCTCCCAGATAGCCGACAAAGT

mPGEs-1	AGGGTGCCATGTTTTCAGAG	CAGTCTTTGGAGGAGCCAAG

EP1	AGCAGATGTGAGGGCAGAGT	GCCTCATCCACTAGGCTCAG

EP2	CCTGGCCATTATGACCATCAC	TCGGGAAGAGGTTTCATCCA

EP3	GTCTAGGCTTGCTGGCTCTG	TGCGTCTTGCATTGCTCTAC

EP4	ACGCGGGCTTCAGTTCCT	CGCACACCAGCACATTGC

BLT1	AGGATGCAGAAACGCTCTGT	CGGTCCAGGCTCATGATAGT

cys-LT 1	GCCTCACCACCTATGCCTTA	CCTGTGGAGGGCTCAAAACAT

Fcγ receptor	CCTGCAGAGTCCTGGCTTAC	CTGATGACTGGGGACCAAAT

### Statistical analysis

Statistical significance was analyzed using Prism 3.0 statistical program (GraphPad Software). Comparisons between two experimental groups were performed with Student's *t *test. Comparisons among three or more experimental groups were performed with ANOVA and a *post hoc *Bonferroni's test to determine significance. A value of *p *< 0.05 was considered significant

## Results

### Defective phagocytosis and proinflammatory cytokine production from young AMs

**Figure 1:**
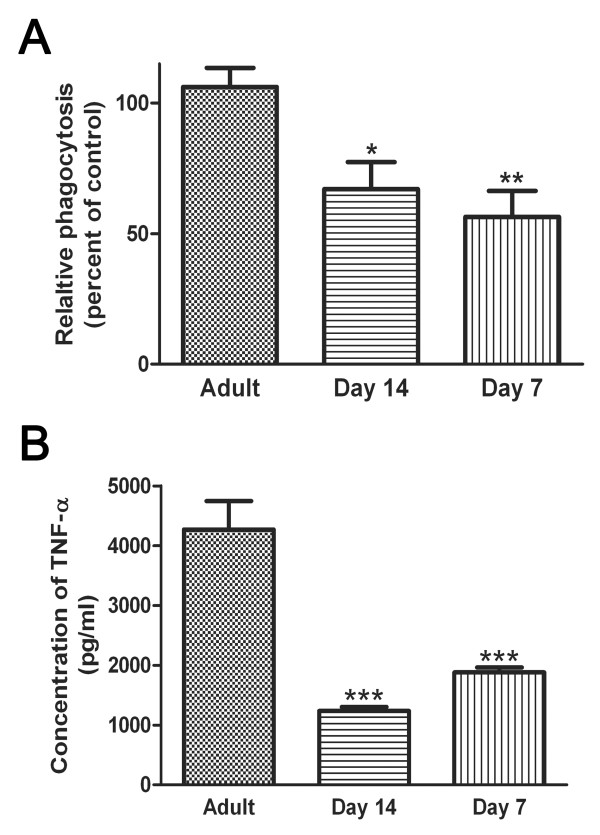
**Decreased phagocytosis and TNF-α production from young AMs compared to Adult AMs**. AMs were harvested from adult (~10 weeks old) and young (day 7 and 14) rats and allowed to adhere overnight. A. AMs were given an opsonized target and the amount of ingestion of the target was quantified after 90 mins. Data represents the mean ± SEM from 4 independent experiments preformed in quadruplicate (*p < 0.05 and **p < 0.01 when compared to adult AMs). B. Supernatants were collected from AM cultures and the amount of TNF-α was measured by a specific EIA. Data shown are representative of three independent experiments preformed in triplicate (***p < 0.001 when compared to adult AMs).

Alveolar macrophages are the cell responsible for identification and clearance of pathogens from the lung. To determine if the increased susceptibility in young rats was due to a defect in these cells, AMs were harvested from adult, day 14 and day 7 rats. We show that young AMs were defective in their ability to ingest an opsonized target (Figure 1A). In addition, AMs from young rats (day 14 and 7) made reduced amounts of the pro-inflammatory cytokine TNF-α at baseline (Figure 1B). Interestingly, when AMs from both young and adult rats were maximally stimulated with LPS, there was no difference in their ability to produce TNF-α (data not shown).

### Alveolar macrophages from young rats produce a skewed eicosanoid profile

**Figure 2:**
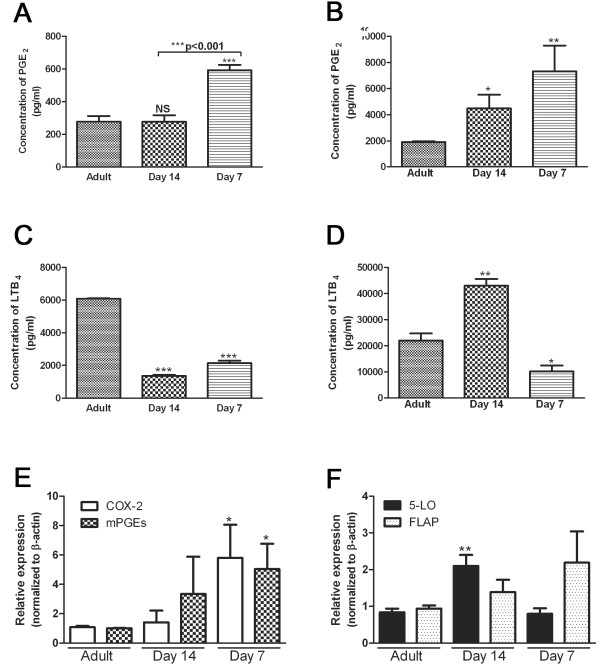
**Dysregulated eicosanoid production and expression of enzymes and proteins responsible for eicosanoid generation from young AMs compared to adult AMs**. AMs were harvested from adult (~10 weeks old) and young (day 7 and 14) rats and allowed to adhere overnight. A. and B. Supernatants were collected from AM cultures that were either unstimulated (A) or stimulated with LPS (B, 500 ng) for 24 h. The amount of PGE_2 _was quantified by specific EIA. C. and D. Supernatants were collected from AM cultures either with media for 24 h (C) or with Ca^2+ ^ionophore A23187 (D, 5 μM) for 1 h. The amount of LTB_4 _was quantified by specific EIA. E. and F. RNA was extracted from the cells, converted into cDNA and the relative expression was determined using semi-quantitative real-time PCR. The primers for specific genes of interest are located in Table 1. For each sample, the expression of the gene of interest was normalized to β-actin and the average of the adult AMs was set to 1. For EIA, data shown are representative of three independent experiments preformed at least in triplicate. For real time PCR experiments, data represents the mean ± SEM of pooled samples from 3 independent experiments. (*p < 0.05, **p < 0.01, and p < 0.001 when compared to adult AMs).

There have been numerous diseases and conditions in which the dysregulation of eicosanoids directly influences host defense functions [11]. AMs were harvested from young and adult rats and the amount of eicosanoids produced were measured at baseline and when maximally stimulated with LPS (to stimulate PGE_2_) or with Ca^2+ ^ionophore A23187 (to stimulate LTs). AMs from day 7 rats made significantly more PGE_2 _at both baseline and when maximally stimulated (Figure 2A and 2B). Unstimulated young AMs (both day 14 and day 7) made significantly less LTB_4 _than adult AMs (Figure 2C). However, when maximally stimulated with Ca^2+ ^ionophore, day 14 AMs make more LTB_4 _whereas day 7 AMs still produce less LTB_4 _(Figure 2D).

### Dysregulation of enzymes and proteins associated with eicosanoid production

There are several enzymes and proteins responsible for the intracellular generation of PGs and LTs. In AMs from day 7 rats, we observed a 5.8-fold increase in mRNA encoding cyclooxygenase (COX)-2 and a 5.0-fold increase in mPGE synthase-1, two inducible enzymes responsible for PGE_2 _production (Figure 2E). In addition, there was no difference in expression of COX-1, the enzyme responsible for basal PGE_2 _production, in young AMs (data not shown). Despite the decrease in LTB_4 _release by day 7 cells, there was no difference in the expression of 5-lipoxygenase (5-LO) (Figure 2F). Interestingly, there was actually an increase in the expression of 5-LO in day 14 AMs. There was no significant difference in the mRNA expression of FLAP (5-lipoxygenase activating protein), an accessory protein necessary for LTB_4 _generation, in the young AMs compared to adult (Figure 2F).

### Increased sensitivity of young AMs to in vitro PGE_2 _stimulation

**Figure 3:**
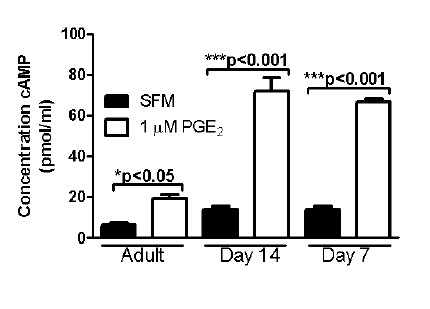
**Elevated cAMP and skewed PDE isoform expression in neonatal macrophages**. AMs were harvested from adult ((~10 weeks old) and young (day 7 and 14) rats and allowed to adhere overnight. A. AMs were stimulated with media or 1 μM PGE_2 _for 20 min before lysing the cells. Intracellular cAMP levels were measured in the whole cell lysate by specific EIA. For cAMP assay, data shown are representative of three individual experiments at least in preformed in duplicate. B. RNA was extracted from the cells, converted to cDNA and the relative expression of each of the PDE3 and PDE4 isoforms was determined using semi-quantitative real-time PCR. For real time PCR experiments, data represents mean ± SEM of pooled samples from 3 independent experiments. (*p < 0.05 and ***p < 0.001 when compared to the unstimulated samples (cAMP assay) and compared to adult AMs (real time PCR)).

The generation of the secondary messenger cAMP has important implications for intracellular signaling cascades and is influenced in opposing fashion by PGE_2 _vs. LTB_4_. Therefore, to determine if young AMs were capable of increased cAMP generation, we measured the in vitro production of this second messenger. We show a slight increase (approximately 2-fold) in the baseline level of cAMP by young AMs (Figure 3A). However, when stimulated with PGE_2_, there was only a 2.2-fold increase in cAMP production by adult AMs compared to a 5.0-fold increase by day 14 AMs and a 4.8-fold increase by day 7 AMs. Thus, in addition to elevated PGE_2 _production and increased PG generating enzymes, we also show that young AMs are more sensitive to PGE_2 _signaling cascades.

### Skewed PDE profile in young verses adult AMs

The level of cAMP in the cell is dependent on the activity of adenylyl cyclase (AC), the enzymes responsible for converting ATP to cAMP, and the activity of PDEs, the enzymes which degrade cAMP [15]. Previous work has shown that PDE3 and PDE4 are the primary isoforms expressed in AMs [16]; therefore, we chose to examine the relative gene expression of each of these enzymes in AMs from young (day 7 and 14) compared to adult AMs (Figure 3B). There was no difference in the expression of PDE4A, PDE4B, or PDE4D between the young and the adult AMs (Figure 3B). However, we do show that specifically young day 7 AMs express high levels of PDE3B and low levels of PDE4C compared to adult AM (Figure 3B). We were not able to detect expression of PDE3A in any of the AMs tested (data not shown).

### No difference in eicosanoid receptor gene expression in young AMs

**Figure 4:**
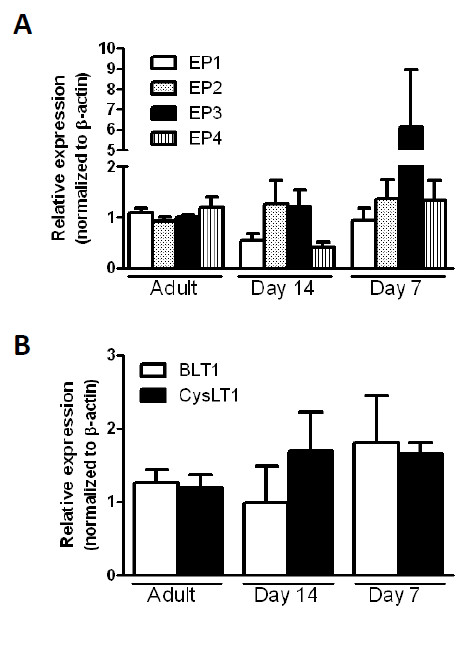
**No difference in eicosanoid receptor expression in young AMs compared to adult AMs**. AMs were harvested from adult ((~10 weeks old) and young (day 7 and 14) rats. RNA was extracted from the cells, converted into cDNA and the relative expression was determined using semi-quantitative real-time PCR. The primers for the specific genes of interest are located in Table 1. For each sample, the expression of the gene of interest was normalized to β-actin and the average of the adult AMs was set to 1. Data represents the mean ± SEM of pooled samples from 3 independent experiments.

To determine if dysregulated eicosanoids influenced expression of their receptors, we measured the amount of GPCRs on young AMs. The relative amount of EP receptor expression was found to be slightly different on young AMs compared to adult AMs. The EP receptor expression profile for adult rat AMs was EP2 > EP1 = EP4 > EP3 compared to young AMs which was EP2 > EP1 = EP3 > EP4. However, when levels of each receptor were normalized and compared, there were no statistically significant changes (Figure 4A). There was a 6.2-fold increase in EP3, the Gi-coupled receptor, expression in day 7 compared to adult, but interestingly, this profile change did not diminish cAMP production in response to PGE_2 _(Figure 3). Although there is a trend toward elevated BLT1 (predominant LTB_4 _receptor on AMs) and cysLT1 (predominant cysLT receptor on AMs) expression on day 7 compared to adult, no statistically significant difference was measured (Figure 4B). Thus, we conclude that overall, there was no difference in receptor expression which could be linked to a functional dysregulation of cAMP in young AMs.

### Young AM phagocytosis is not influenced by modulation of eicosanoids but does correlate with altered expression of Fcγ receptors

**Figure 5:**
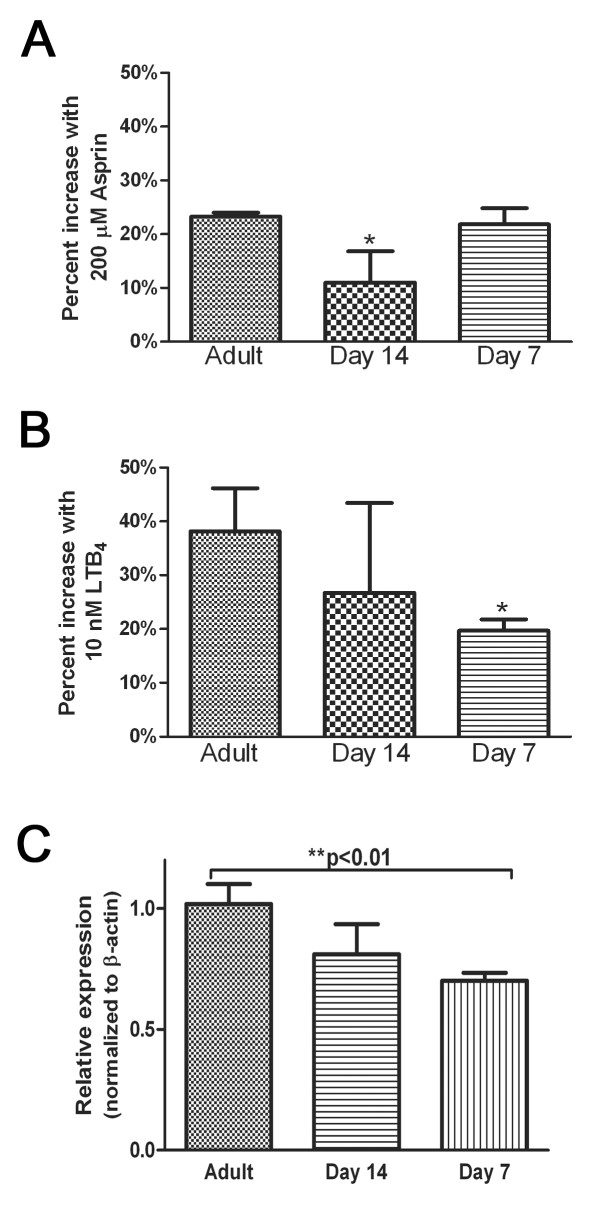
**Modulation of eicosanoids did not alter phagocytosis of young AMs**. AMs were harvested from adult ((~10 weeks old) and young (day 7 and 14) rats and allowed to adhere overnight. A and B. AMs were either untreated or treated with 200 μM aspirin (A) or 10 nM LTB_4 _(B) for 20 min before the addition of an opsonized target. The amount of ingestion of the target was quantified after 90 mins. For phagocytosis assay, data are presented as the percent of increase above baseline phagocytosis for each age group and shown are the mean ± SEM from 3 independent experiments preformed in triplicate. C. RNA was extracted from the cells, converted into cDNA and the relative expression was determined using semi-quantitative real-time PCR. For each sample, the expression of Fcγ receptor was normalized to β-actin and the average of the adult AMs was set to 1. For real time PCR, data represents the mean ± SEM of pooled samples from 3 independent experiments (**p < 0.01 and ***p < 0.001 when untreated group).

To determine whether the increased PGE_2 _or decreased LTs contributed to defective AM function, we incubated AMs from adult or young animals with either aspirin (to inhibit PGs) or exogenous LTB_4_. As expected, treatment with aspirin or exogenous LTB_4 _significantly augmented the phagocytic ability of the adult AMs (Figure 5A and 5B). However, neither treatment was able to enhance the phagocytic ability of young AMs from either age group, suggesting that eicosanoid imbalances were not responsible for the functional defects.

To determine whether differences in phagocytosis of IgG-opsonized targets were due to a differential expression profile of phagocytic receptors, the expression of the Fcγ receptor (CD64) was examined in AMs from young and adult rats. Day 7 young AMs had significantly less Fcγ receptor when compared to adult AMs (Figure 5C).

## Discussion

These studies examined the relationship between macrophage host defense and eicosanoids during the neonatal period. We demonstrate that AMs from young animals (day 7 and day 14) have an impaired ability to phagocytose opsonized targets and produce reduced amounts of TNF-α at baseline. These studies also show increased expression of enzymes responsible for PGE_2 _generation, but no change in EP receptor expression on young macrophages. In addition, there was increased expression of PDE3B and decreased expression of PDE4C in young (day 7) AMs compared to adult AMs. Although there was increased production of the immuno-inhibitory eicosanoid PGE_2_, increased responsiveness to PGE_2 _and decreased production of the immuno-stimulatory eicosanoid LTB_4 _in young AMs, these changes did not influence the functionality of the neonatal AMs. Rather, a possible explanation for the discrepancy between eicosaniod production and phagocytic ability could be the decreased expression of Fcγ receptors on young (day 7) AMs. These results advance the current understanding of the role of lipid mediators in the functionality of neonatal AMs. Previously it was assumed (by analogy to adult studies) that the elevated production of PGE_2 _by neonatal AMs was responsible for their impaired innate immune functions. Our results demonstrate that this is not true and provide an important framework for future therapeutic approaches to treating babies with severe respiratory infections. Our work suggests that neither COX inhibitors nor LT receptor agonists will be effective therapies in this patient population.

Part of the confusion associated with development literature is the definition of 'young' verses 'old' as well as the type of model system employed. In some instances, 'young' refers to animals that are still nursing and only hours or days old [3-6,17]; however in other cases 'young' refers to animals that are already weaned (4-6 weeks old). In this study we limited our focus to 'young' animals that are still nursing. The majority of the studies have been performed using rodent model systems; however some work has been done using monkeys [5] or cattle [12]. Although rodent studies are the simplest and most cost effective system to employ, we recognize that translating the data to humans is difficult due to differences in life span and development [18]. Previous work has shown that at birth, newborns have decreased opsonins in their circulating blood compared to adults which could diminish innate immune responses [19]; however this was normalized at 3 months of age. Based on previous calculations [20], we believe that our rat model reflects the > 3 month age of a human neonate. Thus, we wanted to determine if there was an intrinsic defect in AM function even in the situation where the AMs are presented with equivalently opsonized particles. Additionally, opsonized phagocytosis by neonatal AMs is an understudied area of investigation.

There have been conflicting reports regarding production of extracellular mediators during the neonatal period. Previous work has shown that AMs from both humans (< 2 years of age) [2] and newborn animals [21] produce less TNF- α. However, other studies show that neonatal AMs make similar amounts of pro-inflammatory cytokines such as TNF-α, IL-1 IL-6, IL-12, and IL-18 and chemoattractant cytokines such as MIP-1α and MCP-1 [22,23]. Additionally, elevated production of the immunosuppressive cytokine IL-10 was found in newborn AMs [24]. Previous work has shown that PGE_2 _can inhibit TNF-α [25] and increase IL-10 [26] in macrophages, therefore we investigated whether there were differences in the production of this eicosanoid. Our data show that young AMs produce increased PGE_2. _We were able to demonstrate that the increased synthesis of PGE_2 _in young AMs correlates with increased expression of the PGE_2 _synthetic enzymes, COX-2 and PGEsyn-1.

As expected this enhanced PGE_2 _production correlated with inhibition of TNF-α and diminished phagocytosis. Surprisingly, we discovered that the inhibition of PGs did not improve phagocytosis in the young AMs, but aspirin treatment did enhance adult AM phagocytosis. This result was quite unexpected and highlights a unique difference in young versus adult AMs. Similarly, young AMs produce decreased amounts of the pro-phagocytic leukotriene, LTB_4_. The mechanistic basis for decreased LTB_4 _expression is not clear as it did not correlate precisely with expression of 5-LO or FLAP. Again, addition of exogenous LTB_4 _had no effect on phagocytosis by young AMs. These data highlight the unresponsiveness of young AMs to exogenous factors (both positive and negative) that normally modulate phagocytosis in adult AMs. It will be important to determine whether this phenotype is specific to opsonized phagocytosis only, or if it also extends to non-opsonized phagocytosis in future experiments.

To determine whether the unresponsiveness of young AMs was due to receptor differences, we analyzed expression of the 4 EP receptors and also that of the main LTB_4 _receptor (BLT1) and the main cysteinyl leukotriene receptor (Cys LT1) in young and adult AMs. While there were slight differences in expression profiles noted, none could explain the differential responsiveness of young versus adult AMs. One result which was surprising however, was that despite similarities in EP2 expression in adult and young AMs, the response of young AMs to PGE_2 _stimulation was significantly enhanced as evidenced by the increased generation of cAMP in these cells. Since the receptor profiles suggested that this was not due to increased EP2, we next looked to determine whether differential expression of PDEs could explain why cAMP would accumulate to higher levels in young AMs. We found that young AMs have higher expression of PDE3B and low levels of PDE4C compared to adult AM. Previous work has shown that PDE4 specific inhibitor suppresses TNF-α whereas the PDE3 specific inhibitor did not [27]. Therefore, we speculate that the lack of PDE4C allows for the increased accumulation of cAMP thus leading to decreased levels of TNF-α and impaired opsonized phagocytosis. However, further work will need to be done using PDE inhibitors (such as IBMX and rolipram) to further dissect the mechanism for elevated cAMP response and decreased Fc-mediated phagocytosis in neonatal macrophages.

In addition to the cAMP differences, our work suggests that the opsonized phagocytic defect in young AMs may also relate to diminished Fcγ receptor expression. Expression of Fcγ receptor was significantly decreased in day 7 AMs and trended towards a decrease in day 14 AMs. Previous work has shown that neutrophils and monocytes isolated from neonates have decreased Fcγ receptor expression [28]. However, to our knowledge this is the first report that AMs isolated from neonates also have decreased Fcγ receptor expression. Although our works shows there to be differences in the mRNA expression of CD64 (FcRI), we recognize that there are several other molecules important for opsonized phagocytosis by macrophages including: FcRII (CD32), FcRIII (CD16) and CD14. Additional work needs to be done to examine the cell surface expression and contribution of each of these molecules from the various age groups to determine the impact on the phagocytic ability of these cells.

In additional to differences in the expression of receptors, it is also possible that, the inhibitory signaling machinery downstream of EP2 may be inappropriately coupled to Fcγ in neonates. This may explain why aspirin treatment did not benefit phagocytosis in young AMs. In adult cells, PGE_2 _can inhibit opsonized phagocytosis via activation of phosphatase and tensin homolog deleted on chromosome 10 (PTEN) [29]. Thus, it is possible that PTEN activation is defective in neonatal cells. Alternatively, activation of downstream cAMP effector molecules, such as protein kinase A (PKA) and exchange protein activated by cAMP (Epac) may be impaired in neonatal AMs. Future studies will be needed to determine whether the intracellular signals distal to cAMP are altered in neonates versus adults.

Although differences in Fcγ receptor expression likely account for the defective phagocytosis phenotype in vitro, we speculate that AM dysregulation may have further important implications in host defense functions of neonates in vivo. It is possible that the skewed eicosanoid and cytokine profile of young AMs may influence the functionality of other host defense cells in vivo, such as neutrophils. Previous work has shown that PGE_2 _can also inhibit host defense functions of neutrophils [30]. Prior studies have found a decrease in the total number of neutrophils recruited to the lungs after an infection in young animals [23]. Our findings of decreased LTB_4 _synthesis by young AMs may explain a defect in neutrophil recruitment in vivo. Thus, future studies will also examine the role of lipid mediators in regulating host defense function of neutrophils during development.

## Conclusions

Taken together, our data confirm the observation that neonatal AMs have defective host defense functions and skewed eicosanoid synthetic patterns. This work provides new insight into the defective phagocytosis which characterizes neonatal AMs. The defect in phagocytosis can be explained in part by diminished Fcγ receptor expression, but it is not yet clear why young AMs are functionally unresponsive to manipulation of prostaglandins and leukotrienes These results suggest that neonatal AMs have additional, as yet unidentified, mechanisms which limit their host-defense and make them insensitive to pro- and anti-phagocytic effectors. These studies highlight unique differences in young and adult AMs and indicate that therapeutic strategies to block PG production or enhance LT signaling to improve AM function may be ineffective in very young children. Studies of differentially regulated genes in neonatal and adult macrophages may uncover additional pathways for further testing.

## List of Abbreviations

(5-LO): 5-lipoxygenase; (AM): alveolar macrophage; (BLT): B leukotriene type; (cAMP): cyclic AMP; (COX): cyclooxygenase; (cysLT1): cysteinyl LT type 1; (EP): E prostanoid; (5-lipoxygenase activating protein): FLAP; (GPCRs): G-protein coupled receptors; (LT): leukotriene; (PDE): phosphodiesterase; (PG): prostaglandin; (RBCs): red blood cells; (TNF): tumor necrosis factor- α.

## Competing interests

The authors declare that they have no competing interests.

## Authors' contributions

MNB performed all the experiments, analyzed the data, and wrote the manuscript. MPG and BBM participated in the design of the experiments and manuscript revision. All authors read and approved the final manuscript.
